# The efficacy and accuracy of 3D-guided orthodontic piezocision: a randomized controlled trial

**DOI:** 10.1186/s12903-023-02902-6

**Published:** 2023-03-28

**Authors:** Omar Gibreal, Yasser Al-modallal, Ghiath Mahmoud, Ahmad Gibreal

**Affiliations:** 1 Oral and Maxillofacial Surgery Department, University of Damascus Dental School, Damascus, Syria; 2 Department of Oral and Maxillofacial Surgery, University of Damascus Dental School, Damascus, Syria; 3 Department of Orthodontics, University of Damascus Dental School, Damascus, Syria; 4 Department of Otorhinolaryngology, University of Damascus Medical School, Damascus, Syria

**Keywords:** Severe orthodontic crowding, Alignment and leveling, Flapless piezocision, 3D surgical guide, Acceleration of teeth movement

## Abstract

**Background:**

No randomized controlled trial (RCT) has studied the accuracy of surgical guides used in terms of orthodontic treatment acceleration. Therefore the aim of this trial was to assess computer-guided piezocision-based orthodontic.

**Materials and methods:**

Thirty-two patients with severely crowded upper anterior teeth were enrolled and randomly allocated to either the experimental group (ExpG) or the control one. Subjects of the ExpG received three-dimensional (3D) guided piezoelectric corticotomies on the buccal alveolar bone of the anterior region. Five piezocision cuts were properly performed between each anterior teeth and the adjacent in virtual models. Surgical guides were designed and 3D-printed with preplanned slots that guide gingival and then piezoelectric incisions. The patients underwent Cone-Beam Computed Tomography CBCT before and immediately after surgery. Thus, Predesigned piezocisions were compared to the actual ones in attempt to measure three dimensional deviations of the applied peizocisions.

**Results:**

Ninety-six severe maxillary dental crowding were assigned for eligibility, 40 of them met the inclusion criteria. Thirty-two participants were randomly allocated to the trial`s groups. No patient was lost to follow-up neither from the control nor the experimental group. Overall alignment time (OAT) was reduced by 53% in the experimental group compared to the control group. The mean of the 3D deviation of the surgical guide was 0.23 mm (standard deviation 0.19 mm).

**Conclusion:**

The values of the surgical guide deviation was nearly null, which confirms that this innovative technique is clinically applicable. Furthermore, this technique was impressively effective in accelerating orthodontic tooth movement.

**Trial registration:**

This trial was registered at The ISRCTN registry (ID: ISRCTN65498676 Registration date: 07/04/2021).

## Background

Traditional surgical approaches have been approved to be effective techniques in attempt to accelerate orthodontic teeth movement, but they were characterized by presumed levels of pain [1, 2]. According to studies, pain was listed as the worst undesirable complaint during orthodontic treatments [3]. Moreover, extensive orthodontic treatment period of time may lead to several associated complications, such as gingival effects, caries and root resorptions, in addition to the accompanying pain and discomfort recorded throughout treatment stages [4]. Severe crowding is considered one of the most important and most common types of malocclusion, which concerns both patients and orthodontists [5]. However, The acceptance of conventional corticotomy-assisted orthodontics among patients and orthodontists was generally low, mainly because of postoperative discomfort,complications and the invasive nature of these procedures [6]. Therefore, Flapless piezocision, as an alternative to traditional corticotomy, which was described first by Dibart S et al. [7], has been utilized in orthodontic field recently [8]. Although the piezocision procedure is considered noninvasive surgical technique and had a high rate of acceptance among patients, more high-quality RCTs employing three-dimensional x-ray methods in order to guide these Flapless piezocisions are required [9, 10].

In the currently available literature, no randomized controlled trial (RCT) has studied the accuracy of surgical guides used in terms of orthodontic treatment acceleration yet. Therefore the aim of this trial was to assess 3D piezocision surgical guide efficacy in overcoming the disadvantages of previous blind flapless cortectomies that were used in the orthodontic field.

## Materials and methods

### Study design and registration

**Fig. 1:**
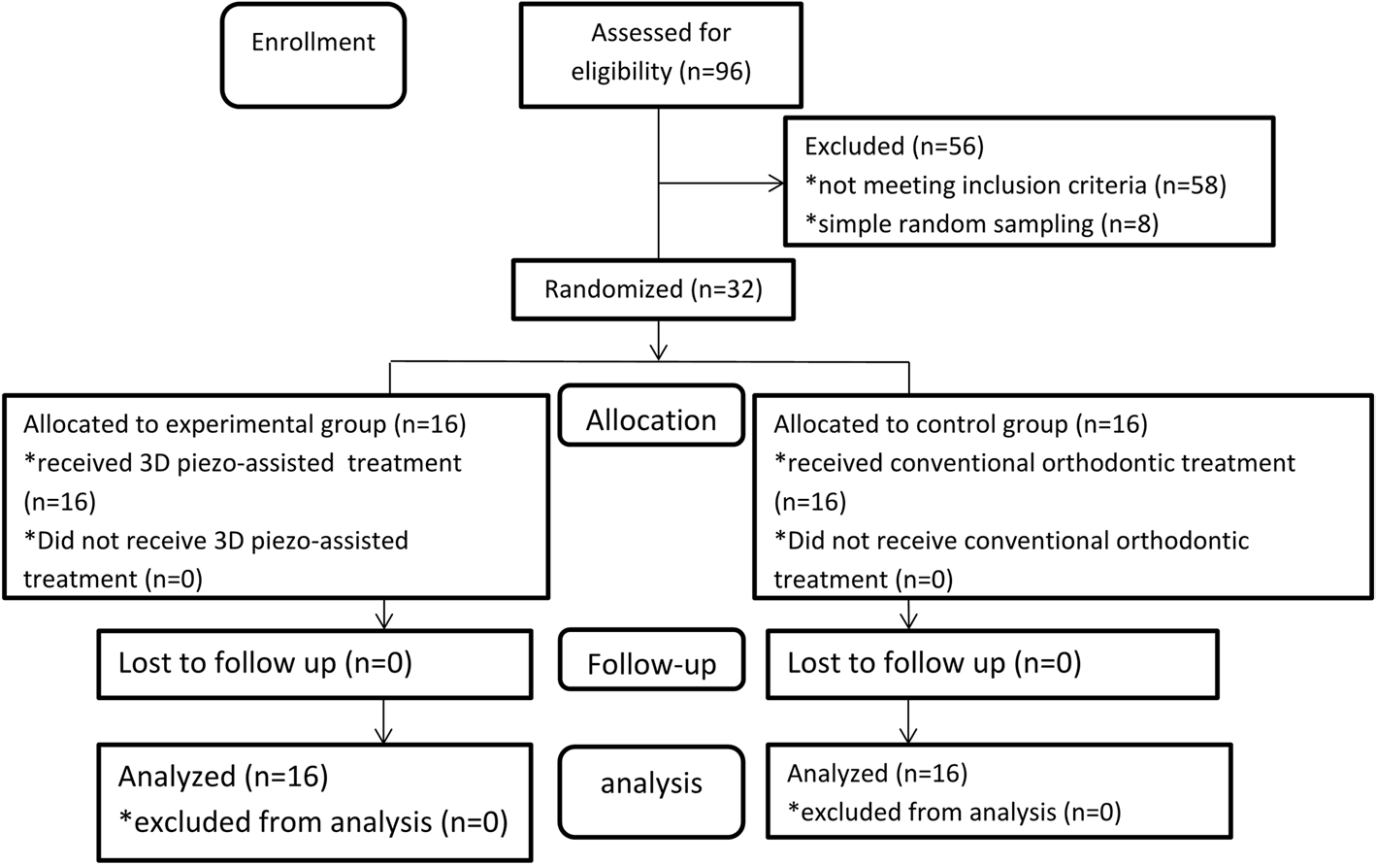
CONSROT 2010 flow diagram of patients’ recruitment and follow-up

This trial was conducted based on the guidelines of Consolidated Standards of Reporting Trials guidelines (CONSORT), where all procedures were performed as were priorly scheduled [11] (Fig. 1). Patients attending the Department of Orthodontics at Damascus University, Dental School, were examined between 25^th^ April 2021 and 30^th^ November 2022. The present study was recorded at The ISRCTN registry (ID: ISRCTN65498676 Registration date: 07/04/2021) retrospectively, i.e., the registration was done after the onset of this trial. This two-arm, parallel-group, single center randomized clinical trial protocol was approved by the Local Research Ethics Committee Approval which was acquired from the University of Damascus (UDDS-588-2018GD/SRC-57782), and the funding for this trial was received from the Postgraduate Research Budget at Damascus University (Ref no: 34627726781JHM).

### Sample size calculation

Sample size was calculated using the G*power 3.1.7 software presuming that a reduction of 30 per cent in total treatment duration could be evidenced with a power of 80 per cent at the 5 per cent significance level. The least clinically significant difference in the time needed for leveling and alignment of severely crowded incisors was assumed to be 52.8 days depending on a previous trial showing that anterior teeth alignment took a mean of 132 days with a standard deviation 39 days [12]. Thus, a sample of 30 patients was required for both groups. To account for possible withdrawal, the final sample size for the study was set at 16 patients per group, yielding a total of 32 patients.

### Patients’ recruitment and eligibility criteria

After clinical examination of 96 patients at the Department of Orthodontics at University of Damascus Dental School, it was found that 40 individuals matched the inclusion criteria. All patients received sufficient explanation about the orthodontic and surgical steps in this trial, and then out of the participants who agreed to take part in this study, 32 were randomly recruited. Information sheets were provided to all selected patients; then, informed consent forms were obtained. The inclusion criteria were: (1) adult ASA I and II patients (I: Normal healthy patient; II: Patients with mild systemic disease) [13] within an age range 18–26 years, (2) maxillary severe crowding (> 6 mm) demanding extraction of the first premolars, (3) completion permanent dentation (except of third molars), (4) Little’s Index range was 10–13 mm, and (5) normal maxillary incisors inclination. The exclusion criteria were: (1) any disease impacting orthodontic movement, (2) medical conditions that would affect tooth movement (corbcosteroid treatments, NSAIDs consumpbon, bisphosphonates, hyperparathyroidism, osteoporosis, uncontrolled diabetes), (3) inadequate oral health, and (4) contraindication to oral surgery (medical–social–psychological).

### Randomization, allocation concealment, and blinding

Subjects were assigned into two parallel groups with a 1:1 allocation ratio by computer-generated list of random numbers. Allocation series was hidden using numbered, opaque, sealed envelopes which were opened at the premolars extraction session. First group received 3D guided piezocisison-assisted orthodontic treatment, while the second group received conventional one. Random allocation sequence generation, patient assignment to interventions,the allocation concealment and outcomes processing were assigned to one of the academic stuff at the Department of Oral and Maxillofacial Surgery(not involved in this research).However, Blinding of personnel and participants was not viable.

### Orthodontic procedures

Orthodontic treatment using traditional metal brackets (Master Series®, American Orthodontics, Sheboygan, Wisconsin, USA), with a 0.022-inch slot high and MBT prescription were used. Then the brackets were bonded after 7 days of the first upper premolars extraction. In both groups, the archwire sequence was as follows: 0.012-inch Nitinol (NiTi), 0.014-inch NiTi, 0.016-inch NiTi, 0.016 × 0.022-inch NiTi, 0.017 × 0.025-inch NiTi, and finally 0.019 × 0.025-inch stainless steel (SS) (American Orthodontics, Sheboygan, Wisconsin, USA) [5]. Replacing wires was accomplished when the used wire became neutral with the ability to insert the next wire without applying exaggerated force. Leveling and alignment was considered finished when LII was less than 1 mm, indicating complete alignment of the anterior teeth and the final archwires were easily and passively inserted into all brackets [14].

### 3D piezocision surgical procedure

**Fig. 2:**
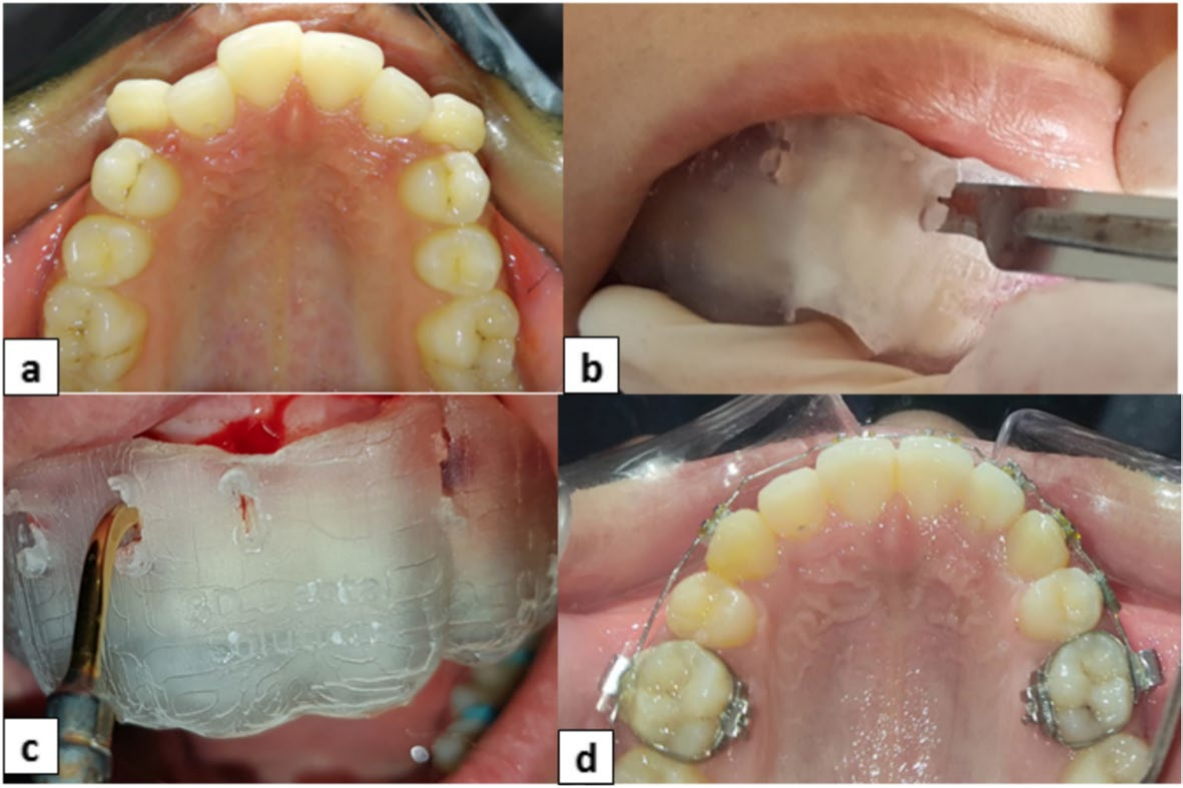
**a** pre-treatment, **b** guided incisions, **c** guided Piezocision, **d** post-treatment

In the experimental group, piezocision surgery was performed on the same premolars extraction day to minimize the number of anesthesia depending sessions in terms of patients’ comfort and satisfaction. The patient was requested to rinse with 0.12% chlorhexidine gluconate (Oral-B, Procter & Gamble Company, USA) before applying the surgical intervention. The surgical procedure was performed, where 3 mm-deep and 5 to 8 mm-long incisions were conducted using a Piezosurgical micro-saw with a BS1 cutting tip (Implant Center™ 2, Satelec, France) (Fig. 2). Patients were asked to follow a soft diet for 2 days after the piezocision and apply mouth rinse for a week [8].

**Fig. 3:**
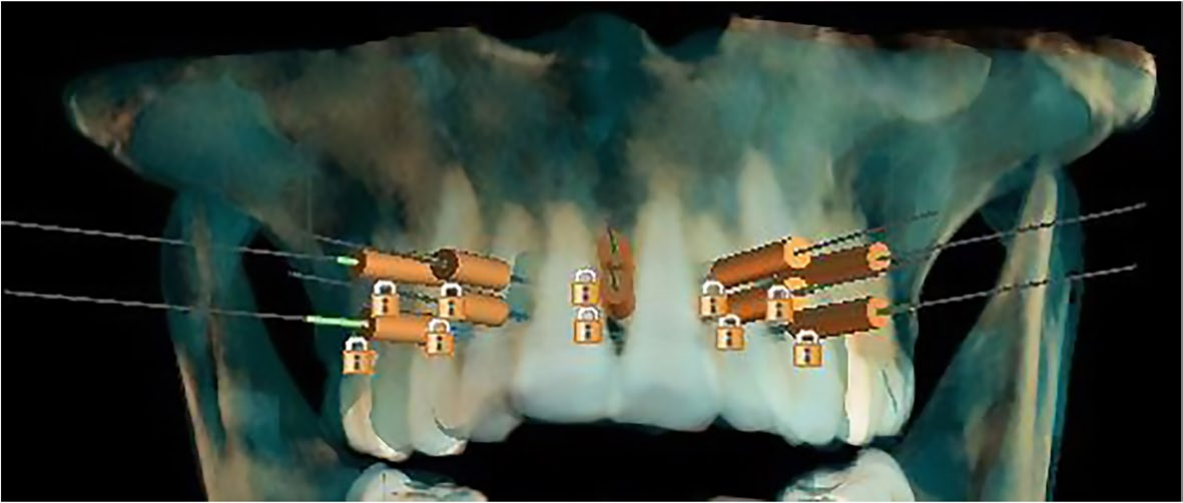
Virtual designing

**Fig. 4:**
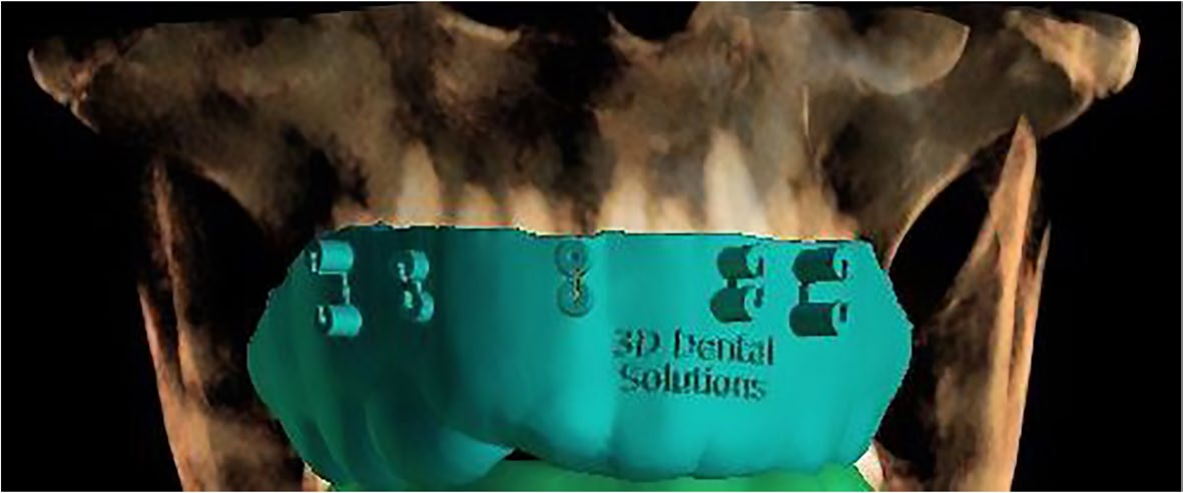
Designed surgical guide

All of the above-mentioned aspects were pre-planned and applied by CBCT imaging and transferred to the casts in order to produce a reliable and precise surgical 3D guide (Figs. 3 and 4).

### Outcome measures

**Fig. 5:**
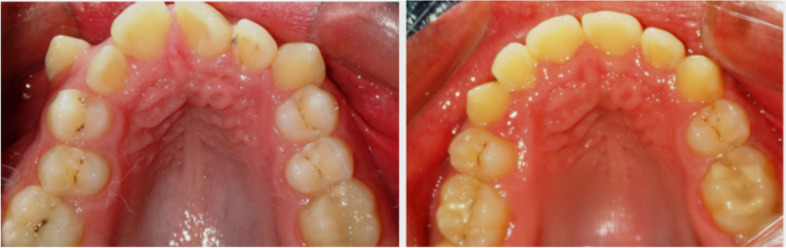
Pre and post treatment control case

The primary outcome measure was the overall alignment time (OAT) required to complete anterior alignment of the maxillary dental arch. Follow-up of this trial was considered finished when the LII was less than 1 mm (Fig. 5).

**Fig. 6:**
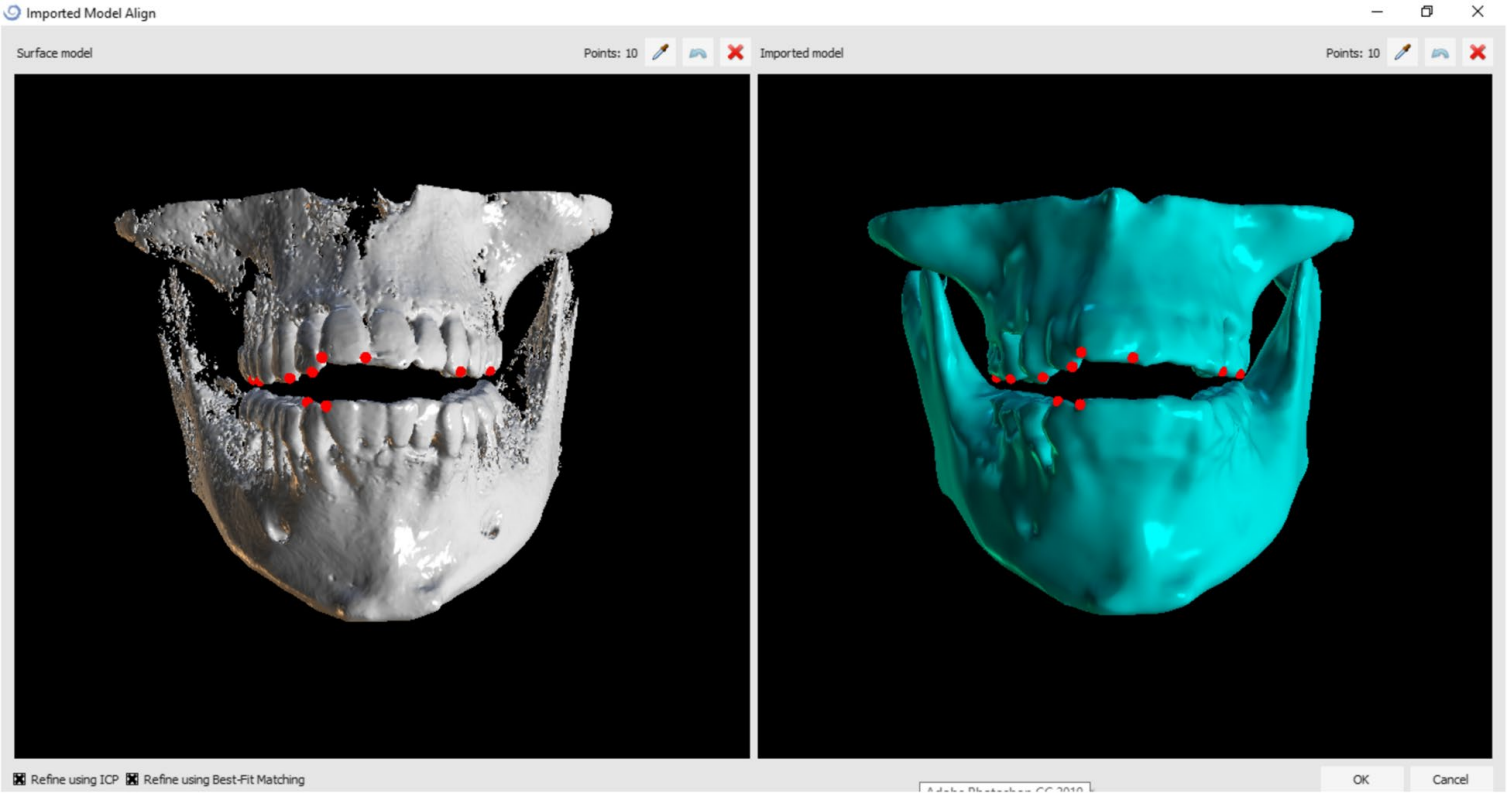
Automated overlapping by the software

The secondary outcome measure was the accuracy of the new surgical guide which assessed by detecting the 3D deviation of the piezocisions. The preoperative (piezocision planning) and postoperative (achieved piezocision position) CBCT scans were performed with the same parameters, then overlapping by a specific algorithm, which allowed the comparison of the virtually planned and the actual piezocision positions, was applied in order to assess the values of deviation (Fig. 6). Three deviation parameters between each planned and placed piezocision were measured. Since the current trial was the first RCT studying the accuracy of 3D piezocision surgical guide, most common method for measuring difference between planned and actual inserted dental implants has utilized.

### Statistical analysis

Parametric tests were used since Anderson–Darling Normality tests showed normal distributions of the collected data. Two-sample t-tests were used to detect significant differences between the two groups regarding OAT.

Single blinding was employed in this trial regarding outcome measure assessment and data analysis. Minitab® program version 17.0 (Minitab Inc., Pennsylvania, USA) was used to perform descriptive and inferential statistics.

## Results

Thirty-two subjects (17 of the 32 patients included in the study were males; 15 were women) participated in this research. The ages of the patients ranged from 18 to 26; the mean age was 20.56 ± 3.71 (Table 1).

**Table 1:** Basic sample characteristics

Group	Gender n (%)	Age Mean (SD)	Maximum age	Minimum age
**Control**	Male 7 (43.75%)	20.22 (2.16)	25	18
	Female 9 (56.25%)			
**Experimental**	Male 10 (62.50%)	21.29 (1.75)	26	18
	Female 6 (37.50%)			
**Total**	32 (100%)	20.56 (3.71)	26	18

There was no patient withdrawal from the study; consequently, all 32 patients were included in the data analysis. The descriptive statistics of the evaluated variables within the groups is summarized in Table 2. The means of piezocision deviation at the buccal-palatal, mesial-distal, coronal apical and three dimensional aspects were 0.15 mm, 0.05 mm, 0.08 mm and 0.23 mm respectively.

**Table 2:** Descriptive statistics of the overall treatment time in the two groups and the values of surgical guide devation (*n* = 16 for each group)

Variable	Group	Mean	SD	Min	Max
OAT	Control	140.1	13.5	95.00	280.00
	Exp	64.8	11.5	42.00	84.00
B-P D	Exp	0.15	0.10	0.00	0.20
M-D D	Exp	0.05	0.03	0.00	0.10
C-A D	Exp	0.08	0.06	0.00	0.15
3D D	Exp	0.23	0.19	0.00	0.40

*OAT* Overall alignment time, *B-P D* Buccal-palatal deviation, *M-D D* Mesial-distal deviation, *C-A D* Coronal-apical deviation, *3D D* Three dimensional deviation, *Exp* Experimental group, *SD* Standard Deviation, *Min*: Minimum, *Max* Maximum

According to the Two sample t test the experimental group was found statistically significant than first group regarding the OAT. The experimental group required less mean treatment time (i.e. 64.8 ± 11.5 days) in the leveling and alignment stage compared to the control group (i.e. 140.1 ± 13.5 days; *P* < 0.0001) with a 53% decrease in the OAT (Table 3).

**Table 3:** The results of significance tests of the observed OAT days) (*n* = 16 in each group)^a^

Group	Mean	SD	*P*-value	95% CI of the difference		Significance of differences
				**Lower bound**	**Upper bound**	
Control	140.1	13.5	0.00001	3.4	5.6	*
Experimental	64.8	11.5				

^*^significant at *P* < 0.0001

^a^Two sample t test

## Discussion

This is the first RCT assessing 3D piezocision-assisted orthodontic in correcting severe crowding of upper anterior teeth in adult patients who underwent extraction-based orthodontic treatment. It was found that 3D guided piezocision accelerated leveling and alignment by about 53%. This could be explained by the regional acceleratory phenomenon (RAP) following the intentional bone injury [15–19]. This result was in accordance with some other recent clinical trials assessing the efficacy of the flapless piezocision technique in accelerating teeth leveling and alignment [8, 9, 12, 20, 21], similar acceleration rates in the piezocision groups were found. None of them used 3D guiding techniques to achieve safe and precise piezoelectric cortectomies except for one [9]. However, this trial performed guided piezocision in accelerating mandibular crowding cases.

In contrast, Uribe et al. found no significant difference between the control and the piezocision groups in terms of the alignment time [22]. The difference between our results and theirs is mainly explained by the dissimilar surgical techniques. They applied less and shallower cortical incisions (4- mm length and 1-mm depth of cortical bone between the mandibular central incisors, and lateral incisors and canines), whereas in the current trial, five cortical incisions in the labial cortical plate( 5- to 8-mm length and 3-mm depth of cortical bone between the six maxillary anterior teeth), which may have reduced the effect of the RAP since the fact of the need for corticotomies deep enough to reach the medullary space to obtain a maximum RAP effect is declared in many previous studies [23–25]

Other RCTs have accomplished regarding the efficacy of piezocision in accelerating orthodontic treatments in combination with Self-ligating brackets recently [18, 26, 27]. Nevertheless, the direct comparison between our findings and those studies is not straightforward since these RCTs have been based on different orthodontic procedures.

Regarding the 3D surgical guide accuracy, the indications of computer-guided piezocision are limited by the maximum deviation detected, which is set as a safety distance of 1.5 mm among anatomical vital structure [28].This rule was applied in the present trial since the results have showed high levels of accuracy for 3D-guided piezocision in order to avoid any possible complications could have happen due to direct contact of the piezoelectric tips with tooth roots or any critical anatomical structure [29]. In this study, the mean deviation value at the three aspects i.e. buccal-palatal, mesial-distal and coronal-apical was 0.15 mm, 0.05 mm and 0.08 mm,respectively, and the mean of 3D-guide deviation was 0.23 mm. As mentioned above, no other RCT has assessed the precision of the surgical guides in terms of accelerating orthodontic teeth alignment. With regard of the current trial, 3D piezocision surgical guide characterize with a high and reproductive results compared with implant surgical guides [30–32]. However, no direct comparison is possible.

Highly accurate recorded outcomes confirm that employing 3D techniques in orthodontic acceleration approaches is an essential aid, especially for flapless techniques since they were mentioned as blind procedures. No significant harms were observed during the entire duration of the study.

### Limitations

The current RCT has some limitations. First, the Hawthorne effect and detection bias are possible to happen due to the inability to blind both patients and the principal researcher. Secondly, lack of previous well designed trials led to the impossibility of direct comparison regarding the guide accuracy. Finally, mensuration of efficacy and accuracy outcomes using other acceleration methods in different malocclusion cases is recommended in forthcoming research.

## Conclusions

The values of the surgical guide deviation was nearly null, which confirms that this innovative technique is clinically applicable. Furthermore, this technique was impressively effective in accelerating orthodontic maxillary teeth alignment by 53%.

## Data Availability

The datasets used and/or analysed during the current study available from the corresponding author on reasonable request.

## Abbreviations

ExpG: Experimental group
LII: Little’s Irregularity Index
3D: Three-dimensional
